# Hyperglycemia and Physical Impairment in Frail Hypertensive Older Adults

**DOI:** 10.3389/fendo.2022.831556

**Published:** 2022-04-14

**Authors:** Antonella Pansini, Angela Lombardi, Maria Morgante, Salvatore Frullone, Anna Marro, Mario Rizzo, Giuseppe Martinelli, Eugenio Boccalone, Antonio De Luca, Gaetano Santulli, Pasquale Mone

**Affiliations:** ^1^ ASL Avellino, Avellino, Italy; ^2^ ASL Caserta, Caserta, Italy; ^3^ Department of Medicine, Einstein Institute for Aging Research, Einstein-Sinai Diabetes Research Center, Fleischer Institute for Diabetes and Metabolism (FIDAM), Albert Einstein College of Medicine, New York, NY, United States; ^4^ Campania University, Caserta, Italy; ^5^ ASL Napoli, Naples, Italy

**Keywords:** aging, blood glucose, cognitive impairment, COPD, diabetes, elderly, gait speed, MoCA score

## Abstract

**Background:**

Frailty is a multidimensional condition typical of elders. Frail older adults have a high risk of functional decline, hospitalization, and mortality. Hypertension is one of the most common comorbidities in elders. Hyperglycemia (HG) is frequently observed in frail older adults, and represents an independent predictor of worst outcomes, with or without diabetes mellitus (DM). We aimed at investigating the impact of HG on physical impairment in frailty.

**Methods:**

We studied consecutive older adults with frailty and hypertension at the ASL (local health unit of the Italian Ministry of Health) of Avellino, Italy, from March 2021 to September 2021. Exclusion criteria were: age <65 years, no frailty, no hypertension, left ventricular ejection fraction <25%, previous myocardial infarction, previous primary percutaneous coronary intervention and/or coronary artery bypass grafting. Blood glucose, Hb1Ac, and creatinine were measured in all patients. Physical frailty was assessed applying the Fried Criteria; we performed a 5-meter gait speed (5mGS) test in all patients.

**Results:**

149 frail hypertensive older adults were enrolled in the study, of which 82 had normoglycemia (NG), and 67 had HG. We observed a significantly slower 5mGS in the HG group compared to the NG group (0.52 ± 0.1 *vs*. 0.69 ± 0.06; p<0.001). Moreover, we found a strong and significant correlation between 5mGS and glycemia (r: 0.833; p<0.001). A multivariable linear regression analysis using 5mGS as a dependent variable revealed a significant independent association with glycemia (p<0.001) after adjusting for likely confounders.

**Conclusions:**

HG drives physical impairment in frail hypertensive older adults independently of DM.

## Background

Frailty is a multidimensional condition typical of elders that determines physical decline. Frail older adults have a high risk of functional decline, hospitalization, and mortality (1–4). Hence, a careful geriatric evaluation is one of the best strategies to obtain an early diagnosis of physical impairment, and managing comorbidities and complications is fundamental to counteract it (5–11). Hypertension is one of the most common comorbidities in elders, affecting endothelial function, leading to oxidative stress, inflammation, and atherosclerosis (12–19).

Hyperglycemia (HG) is frequently observed in frail hypertensive older adults, and we and others have shown that it represents an independent predictor of worst outcomes, even if diabetes mellitus (DM) is not present (20–23). Indeed, HG drives inflammation and oxidative stress, leading to endothelial dysfunction, with a negative impact on frail patients (7, 24–28).

In this context, reaching and maintaining an optimal glycemic control may be crucial to reduce the incidence of functional decline and avoid complications (11, 29–32). On these grounds, we investigated the impact of HG on physical impairment in frail hypertensive older adults.

## Methods

We studied consecutive older adults with frailty and hypertension at the ASL (local health unit of the Italian Ministry of Health) of Avellino and Caserta, Italy, from March 2021 to September 2021.

Inclusion criteria were: Age ≥65 years; frailty; primary hypertension. Exclusion criteria were: Age <65 years; absence of frailty; secondary hypertension or absence of hypertension; previous myocardial infarction, left ventricular ejection fraction <25%, and previous cardiac revascularization.

HG was defined as blood glucose level ≥140 mg/dL according to previous investigations that evaluated HG in complex patients, both diabetic and non-diabetic (33–37), and following ADA recommendations, which refer to this value for hospitalized patients (38) and/or subjects with impaired glucose tolerance (39).

Hypertension was defined as systolic blood pressure (SBP) ≥140 mmHg and/or diastolic blood pressure (DBP) ≥90 mmHg on repeated measurements, or as a previously diagnosed hypertension (40). Blood samples to measure glycemia, HbA1c, hyperlipidemia, and creatinine were taken from all patients. The study was approved by the Campania Nord Ethical Committee. A written informed consent was signed by all patients.

### Assessment of Physical Frailty

A diagnosis of frailty status was made according to the Fried Criteria, as we previously reported (19, 41):

- Weight loss (unintentional loss ≥4.5 kg in the past year);
- Weakness (handgrip strength in the lowest 20% quintile at baseline, adjusted for sex and body mass index);
- Exhaustion (poor endurance and energy, self-reported);
- Slowness (walking speed under the lowest quintile adjusted for sex and height);
- Low physical activity level (lowest quintile of kilocalories of physical activity during the past week).

Frailty was diagnosed with at least 3 criteria out of 5.

A 5-meter gait speed (5mGS) test was performed in all patients, as we previously described (42). 5mGS was advocated as a reliable measure of physical capacity in frail patients with cardiovascular diseases (43). Indeed, this test evaluates lower extremity muscle function, neurological and cardiopulmonary capacity (44, 45).

### Statistical Analysis

Data are presented as mean ± SD or percentage. We developed a dispersion model using Pearson analysis to assess the correlation between glycemia and 5mGS. To explore the impact of comorbidities, we carried out a multivariable linear regression model with a 5mGS test as a dependent variable. All calculations were performed using the software Statistical Product and Service Solutions (SPSS) version 26.

## Results

**Figure 1:**
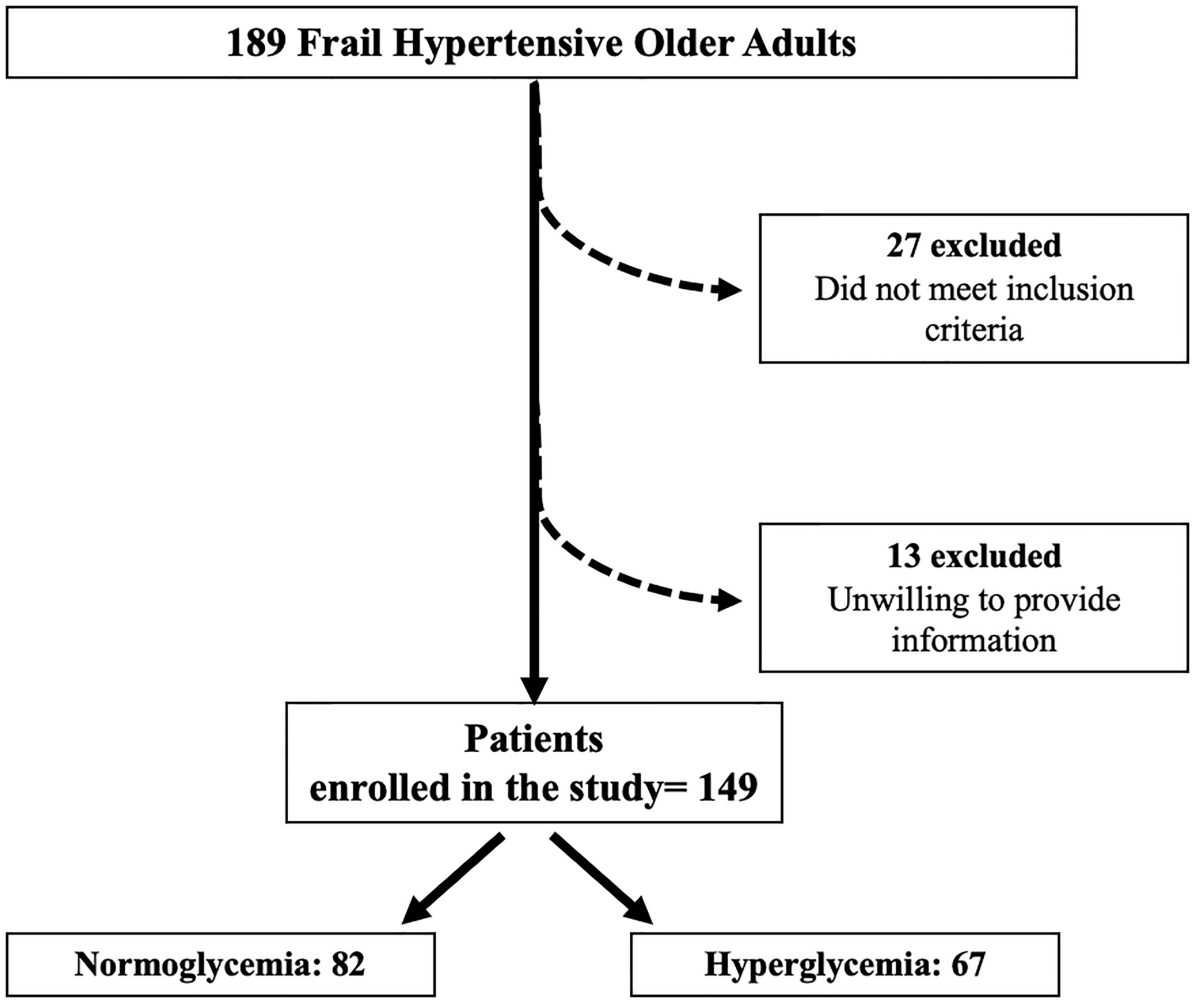
Study flow diagram.

We screened 189 frail hypertensive patients. Since 13 patients did not give their consent and 27 subjects did not meet inclusion criteria, 149 patients were enrolled in the study, of which 82 had normoglycemia (NG) and 67 had HG (**Figure 1**).

**Figure 2:**
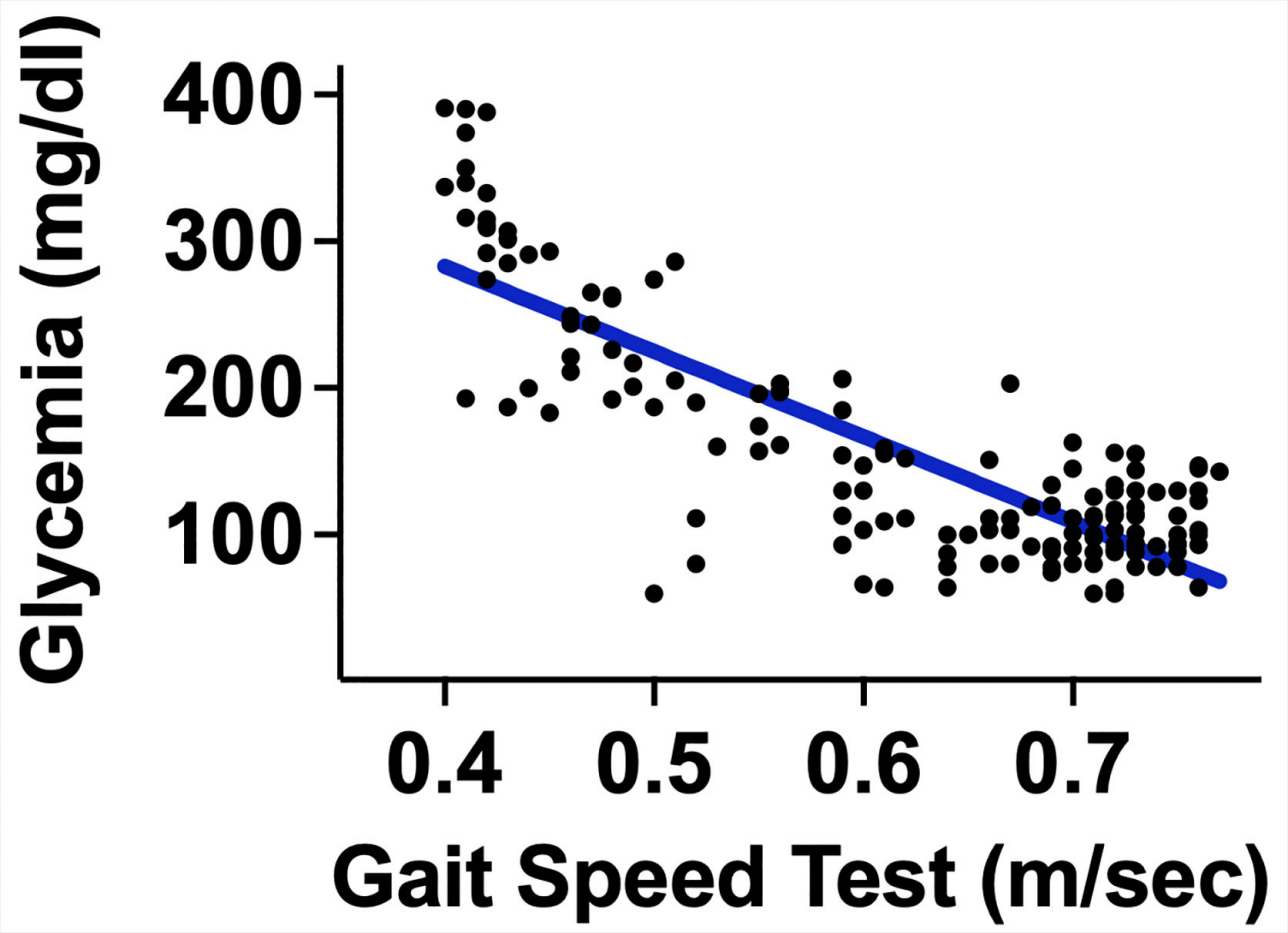
Dispersion model correlating glycemia and 5-meter gait speed.

Patients were similar in age, BMI, sex distribution, and comorbidities (**Table 1**). We found a strong and significant correlation between 5mGS and glycemia (r: 0.833; 95% C.I.: -0.8766 to -0.7765; p<0.001) in all patients (**Figure 2**).

**Table 1:** Clinical characteristics of the patients.

	NG	HG
N	82	67
Sex (M/F)	36/46	29/38
Mean age (years)	84.62 ± 6.1	84.48 ± 6.3
BMI (kg/m^2^)	27.7 ± 1.6	27.9 ± 1.6
SBP (mmHg)	118.7 ± 7.4	119.0 ± 7.8
DBP (mmHg)	79.6 ± 6.7	79.4 ± 6.3
Heart rate (bpm)	87.3 ± 9.8	87.1 ± 8.8
5mGS (m/s)	0.69 ± 0.06	0.52 ± 0.1*
**Comorbidities, n (%)**		
Diabetes	32 (39.0)	54 (80.6)*
COPD	38 (46.3)	33 (49.3)
CKD	39 (47.6)	35 (52.2)
CVD	44 (53.7)	34 (50.7)
Hyperlipidemia	43 (52.4)	37 (55.2)
**Laboratory analyses**		
Plasma glucose (mg/dl)	100.1 ± 19.6	231.5 ± 71.4*
HbA1c, mmol/mol (%)	57 ± 5.5 (7.4 ± 0.5)	58 ± 5.5 (7.5 ± 0.5)
Serum creatinine (mg/dl)	1.1 ± 0.5	1.2 ± 0.5
**Global Cognitive Evaluation**		
MoCA	19.5 ± 3.6	19.1 ± 3.9

Data are means ± SD or n (%). 5mGS, 5 m gait speed; BMI, body mass index; CKD, chronic kidney disease; COPD, chronic obstructive pulmonary disease; CVD, cerebrovascular disease; DBP, diastolic blood pressure; HbA1c, glycated hemoglobin; HF, heart failure; HG, hyperglycemic; MoCA, Montreal Cognitive Assessment; NG, normoglycemic; SBP, systolic blood pressure. *p < 0.001.

**Figure 3:**
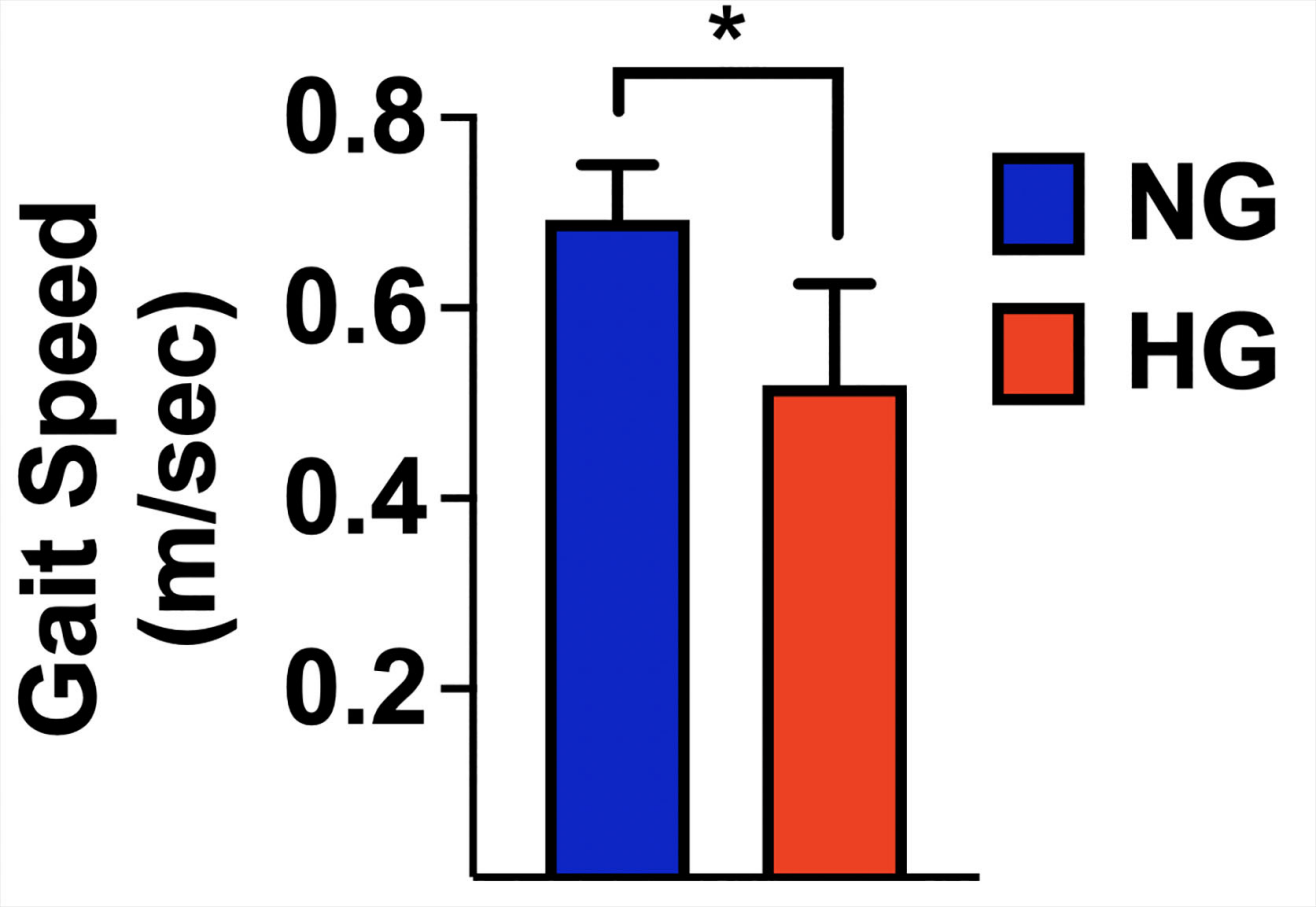
Gait speed measured in normoglycemic (NG) and hyperglycemic (HG) patients; mean±SD; *p < 0.001.

We observed a significantly slower 5mGS in the HG group compared to the NG group (0.52 ± 0.1 *vs*. 0.69 ± 0.06; p<0.001) (**Figure 3**). A multivariable linear regression analysis with 5mGS as a dependent variable (**Table 2**) confirmed the significant impact of glycemia (p<0.001) and revealed also an association with COPD (p: 0.043).

**Table 2:** Linear regression analysis with 5mGS as a dependent variable.

	B	Standard Error	Beta	t	p	95% Confidence Interval	
						Lower Bound	Upper Bound
***Age***	0.002	0.001	0.091	1.312	0.192	-0.001	0.004
***Diabetes***	-0.006	0.017	-0.025	-0.355	0.723	-0.040	0.028
***CVD***	-0.017	0.017	-0.072	-1.042	0.299	-0.050	0.015
***Hyperlipidemia***	0.014	0.016	0.060	0.922	0.358	-0.016	0.045
***CKD***	0.019	0.015	0.080	1.242	0.216	-0.011	0.049
***COPD***	0.029	0.014	0.121	2.038	0.043	0.001	0.058
***Glycemia***	-0.001	0.000	-0.854	-14.672	<0.001	-0.002	-0.001
***Serum creatinine***	-0.026	0.015	-0.124	-1.679	0.095	-0.056	-0.006
***HbA1c***	-0.008	0.011	-0.035	-0.714	0.476	-0.029	0.014
***BMI***	-0.003	0.003	-0.045	-0.957	0.340	-0.011	0.003
***SBP***	0.001	0.001	0.049	0.806	0.421	-0.001	0.002
***DBP***	0.001	0.001	0.036	0.731	0.466	-0.001	0.002
***HR***	-0.001	0.001	-0.074	-1.150	0.136	-0.002	0.000

BMI, body mass index; CKD, chronic kidney disease; COPD, chronic obstructive pulmonary disease; CVD, cerebrovascular disease; DBP, diastolic blood pressure; Hb1Ac: glycated hemoglobin; HR, heart rate; SBP, systolic blood pressure.

## Discussion

Our study indicates that frail hypertensive elders with HG have a significantly lower 5mGS compared to NG subjects. It is important to emphasize the fact that these results refer to a frail hypertensive population of older adults, in which physical performance affects functional decline, loss of independence, and cognitive impairment (30, 46).

Glucose levels may increase the risk of frailty in older adults without DM (31). It is interesting to observe that these findings are independent of a previous diagnosis of DM as well as from HbA1c values. In this scenario, HG drives physical impairment independently of DM and we speculate that glycemic control appears to be the best way to attempt to reverse physical impairment, with or without DM.

Our study does have some limitations. First, the study population is relatively small; second, there is no follow-up. Therefore, further studies are necessary to confirm our results, ideally in large randomized trials. We also reckon that a majority of our study population is represented by women; this finding is in agreement with the REPOSI Study on elderly people (47). Consistent with our observations, HG is associated with the development of frailty and lower extremity mobility limitations in older women (48, 49). Furthermore, a previous study had suggested to consider functionally independent women with osteoporosis and arthritis as a different cluster of frailty (50).

## Conclusions

Taken together, our data indicate that HG drives physical impairment in frail and hypertensive older adults independently from DM and HbA1c values.

## Data Availability Statement

The original contributions presented in the study are included in the article. Further inquiries can be directed to the corresponding author.

## Ethics Statement

The studies involving human participants were reviewed and approved by the Campania Nord Ethics Committee. The patients/participants provided their written informed consent to participate in this study.

## Author Contributions

AP, AL, GS, and PM designed the study, contributed to drafting the manuscript, approved its final version, and made the decision to submit and publish the manuscript. MM, MR, GM, ADL, and PM analyzed data, revised the manuscript’s intellectual content, and approved the final version. EB, SF, and AM acquired the data, revised the manuscript’s intellectual content, and approved the final version. PM is the guarantor of this work and, as such, had full access to all the data in the study and takes full responsibility for the integrity of the data and the accuracy of data analysis. All authors contributed to the article and approved the submitted version.

## Funding

The Santulli’s Lab is supported in part by the *National Institutes of Health* (NIH: R01-HL146691, R01-DK123259, R01-HL159062, R01-DK033823, and T32-HL144456, to GS), by the Diabetes Action Foundation, by the Irma T. Hirschl and Monique Weill-Caulier Trusts (to GS).

## Conflict of Interest

The authors declare that the research was conducted in the absence of any commercial or financial relationships that could be construed as a potential conflict of interest.

## Publisher’s Note

All claims expressed in this article are solely those of the authors and do not necessarily represent those of their affiliated organizations, or those of the publisher, the editors and the reviewers. Any product that may be evaluated in this article, or claim that may be made by its manufacturer, is not guaranteed or endorsed by the publisher.
